# Dual Mutation Events in the Haemagglutinin-Esterase and Fusion Protein from an Infectious Salmon Anaemia Virus HPR0 Genotype Promote Viral Fusion and Activation by an Ubiquitous Host Protease

**DOI:** 10.1371/journal.pone.0142020

**Published:** 2015-10-30

**Authors:** Mickael Fourrier, Katherine Lester, Turhan Markussen, Knut Falk, Christopher J. Secombes, Alastair McBeath, Bertrand Collet

**Affiliations:** 1 Aquaculture and Fish Health, Marine Scotland Science, Aberdeen, United Kingdom; 2 Epidemiology, Norwegian Veterinary Institute, Oslo, Norway; 3 School of Biological Sciences, University of Aberdeen, Aberdeen, United Kingdom; St. Jude Children's Research Hospital, UNITED STATES

## Abstract

In Infectious salmon anaemia virus (ISAV), deletions in the highly polymorphic region (HPR) in the near membrane domain of the haemagglutinin-esterase (HE) stalk, influence viral fusion. It is suspected that selected mutations in the associated Fusion (F) protein may also be important in regulating fusion activity. To better understand the underlying mechanisms involved in ISAV fusion, several mutated F proteins were generated from the Scottish Nevis and Norwegian SK779/06 HPR0. Co-transfection with constructs encoding HE and F were performed, fusion activity assessed by content mixing assay and the degree of proteolytic cleavage by western blot. Substitutions in Nevis F demonstrated that K276 was the most likely cleavage site in the protein. Furthermore, amino acid substitutions at three sites and two insertions, all slightly upstream of K276, increased fusion activity. Co-expression with HE harbouring a full-length HPR produced high fusion activities when trypsin and low pH were applied. In comparison, under normal culture conditions, groups containing a mutated HE with an HPR deletion were able to generate moderate fusion levels, while those with a full length HPR HE could not induce fusion. This suggested that HPR length may influence how the HE primes the F protein and promotes fusion activation by an ubiquitous host protease and/or facilitate subsequent post-cleavage refolding steps. Variations in fusion activity through accumulated mutations on surface glycoproteins have also been reported in other orthomyxoviruses and paramyxoviruses. This may in part contribute to the different virulence and tissue tropism reported for HPR0 and HPR deleted ISAV genotypes.

## Introduction

Infectious Salmon Anaemia Virus (ISAV) is an orthomyxovirus which causes disease in farmed Atlantic salmon (*Salmo salar* L.). Outbreaks have been reported in all the main salmon producing countries where this viral disease has led to high mortality and serious financial losses [1–6]. The virus is enveloped with a genome consisting of 8 single-stranded RNA segments in negative orientation. Segments 5 and 6 encode two surface glycoproteins: the Fusion (F) protein and Haemagglutinin-esterase (HE), respectively.

In the HE, the haemagglutinin function allows ISAV to attach to 4-*O*-Acetylated sialic acid receptors on the surface of host cells [7,8], while the acetyl esterase function destroys the sialic acid bonds promoting virus release [7,8]. The F protein is responsible for virus and host cell membrane fusion that allows the delivery of the viral genetic material into the cell [9]. There is also mounting evidence that both the ISAV HE and F proteins act in a cooperative manner to trigger fusion during the early stage of infection [9,10].

Virulence is a multifactorial trait which depends on various viral functions such as virus receptor binding [11], cellular uptake, replication rate and shedding of new virions [12,13], modulation of host immune response [14,15] and the ability to spread to new hosts [16]. Surface glycoproteins are particularly important for virulence determination in enveloped viruses as demonstrated with mammalian [11,17,18] and avian influenza viruses [19–25]. More specifically, the fusion activity of a virus can influence its uptake and also define its viral cell tropism through the availability of the appropriate activating protease in different tissues or cells [26–28]. A detailed investigation of how introduced mutations influence surface proteins function is essential to elucidate the mechanisms leading to the acquisition of a higher virulence. Such a level of understanding has been achieved using site directed mutation analysis for several viruses such as Newcastle Disease virus (NDV) [29], avian influenza viruses [30], and Measles viruses [31]. In contrast, the HE and F protein of ISAV have only been the subject of few such functional studies [9,10,32,33] and how mutations influence viral infectivity and virulence in general remains poorly understood.

Non-virulent HPR0 variants of ISAV [4,34–38] provide an opportunity to create a comparative model to understand how specific mutations influence surface protein function and virulence in relation to pathogenic strains. These HPR0 variants are characterised by a HE gene carrying a full length Highly Polymorphic Region (HPR), a 35 amino acid (aa) stretch located in the near membrane domain of the HE stalk [34,39–41]. These virus variants have not been associated with disease in Atlantic salmon, are primarily detected in gills by RT-qPCR and sequencing, and have so far not been propagated in tissue culture [6]. Moreover, all currently characterized pathogenic ISAV isolates have deletions in the HPR in relation to the HPR0 genotype [38,39,42]. This led to the classification of ISAV and their virulence based on HPR genotypes [39]. It also gave rise to the hypothesis that virulent ISAV strains may arise from a reservoir of full length non-pathogenic HPR0 through deletion events in the HE gene HPR [4,39,41,43] and potential sequence alterations in the F gene [44]. Recently, we demonstrated that deletions generated within a full length HPR significantly influenced the ability of the resulting mutant HEs to interact with the F protein and trigger fusion [10]. Our work also suggested that the aa composition of the associated F protein was a critical factor influencing the fusion process in conjunction with these deletions in the HE stalk.

The ISAV F is a pH-dependent class I membrane fusion protein which needs cleavage by a host proteolytic enzyme for activation [9,45]. The F protein is generated as a 50 kDa inactive precursor molecule (F0), which once cleaved results in two disulphide linked subunits, F1 and F2 at 30 and 20 kDa respectively [9]. In other viruses, the newly formed amino F1 terminus is usually highly hydrophobic and functions as a fusion peptide which later leads to the merging of the virus and host cell membranes [46]. In ISAV, two potential cleavage sites, a lysine (K) in position 276 and arginine (R) in 267, have been proposed [9], but have yet to be functionally confirmed.

Viral fusion consists of a succession of discrete molecular events involving the combined action of the attachment and fusion proteins [47]. Therefore, the precise location of mutations within surface glycoproteins tends to influence specific steps of the fusion process. In measles viruses, alteration of the attachment protein stalk affected how the H activated the F protein and subsequent fusion [31]. In ISAV, deletions within the HPR were suggested to influence the separation between HE and F proteins with a shorter stalk region resulting in higher fusion levels [10]. Since type I fusion proteins require activation by proteolytic cleavage, mutations in or near the cleavage site can define cleavage specificity and are known to impact cell tropism and the virulence status of influenza viruses [27,48–51] and NDV [46,52–55]. In ISAV, a glutamine (Q) to leucine (L) substitution in position 266 in the F protein was described as an important virulence determinant [44,56,57] and small sequence insertions near the putative cleavage sites have also been reported [36,56,58], which might influence enzymatic cleavage.

This study aimed at understanding how particular HE and F protein mutations influence specific steps of the ISAV fusion process by using surface glycoproteins from a non-virulent HPR0 genotype as a comparative model. The first objective was to functionally identify the actual cleavage site out of the two proposed previously. Secondly, we investigated separately whether single aa substitutions and insertions in the F protein influenced the proteolytic cleavage and fusion activity. Thirdly, a mutated HE with an HPR deletion was combined with these F protein mutants and the effect on F protein activation and proteolytic cleavage specificity assessed under normal culture conditions and following exposure to trypsin and low pH. Finally, fusion activity was also measured following the addition of calcium ionophore A23187, in an attempt to narrow down the potential type of cellular host proteases involved in ISAV fusion protein activation. The potential implications for the virulence of the different ISAV HPR genotypes are also discussed.

## Materials and Methods

### Recombinant plasmids and site-directed mutagenesis

PhrGFP-1 plasmids containing the open reading frame (ORF) from segments 5 (AJ277461) and 6 of the ISAV isolate Nevis 390/98 (AJ276859) were constructed as described previously [33]. Two mutant F proteins harbouring a substitution (R267A and K276A) were generated from the Nevis 390/98 isolate segment 5 in order to identify the true proteolytic cleavage site. These substitutions were generated using a QuickChange II site directed Mutagenesis kit (Agilent) as per manufacturer’s protocol. Primers details and the reference names used throughout this study are provided in Tables 1 and 2. Cloning and purification of mutated plasmids was done as described previously [33].

**Table 1:** Primers used to generate aa substitutions in the Nevis F protein. These mutations aimed at identifying the cleavage site from the two proposed. Mutated codons are in bold and underlined in each DNA sequence.

Name	Position	Mutation	Direction	Primer sequence
F-Mut- R267A	267	Substitution	Fw	GGATGGTCTAAATACAGCTTCAACCTG**GCA**GCATTCCCAGG
			Rev	CCTGGGAATGC**TGC**CAGGTTGAAGCTGTATTTAGACCATCC
F-Mut- K276A	276	Substitution	Fw	CCAGGAGAAGAGTTCATC**GCA**TGCTGTGGATTTACTTTGGGG
			Rev	CCCCAAAGTAAATCCACAGCA**TGC**GATGAACTCTTCTCCTGG

**Table 2:** Primers used to generate a deletion and mutations in the SK779/06 HE and F proteins respectively. Mutated codons are in bold and underlined in each DNA sequence. This table also provides information on the two sequences which were synthesised by Geneart (Invitrogen) and inserted in the F-SK779/06 (in bold and underlined). The position of the HPR deletion generated in the HE is also presented in the first row.

Name	Position	Mutation	Direction	Primer sequence
HE-Mut- Del.	352–371	Deletion	Fw	TATGGGTGTAGCAGGTTTTGGGATT
			Rev	GTGGAGTCGACTTGGTTTG
F-Mut- G252S	252	Substitution	Fw	ACACTAAGAGCC**TCC**CTCGCTAACCAACATGG
			Rev	CCATGTTGGTTAGCGAG**GGA**GGCTCTTAGTGT
F-Mut- Q266L	266	Substitution	Fw	AAATACAACTTCAAC**CTG**AGAGCATTCCCAGG
			Rev	CCTGGGAATGCTCT**CAG**GTTGAAGTTGTATTT
F-Mut- Q266P	266	Substitution	Fw	AAATACAACTTCAAC**CCG**AGAGCATTCCCAGG
			Rev	CCTGGGAATGCTCT**CGG**GTTGAAGTTGTATT
F-Mut- Ins 1	266–267	12 aa insertion		AAC**AAAGGGAAATCAGCTAATGACATTATCTCCGACCAG**AGA
F-Mut- Ins 2	266–267	10 aa insertion		TTC**GGACACTCTGTGCACAAGCTTTCTAACCAG**AGA

Plasmid encoding for the complete ORF sequences of segment 5 (EU118819) and 6 (EU118820) from a Norwegian HPR0 variant SK779/06 [56] were produced by gene synthesis (Geneart, Life technologies) and sub-cloned into phrGFP-1 plasmid using the *Sac*I and *Xho*I sites as described previously [33]. Using the SK779/06 segment 5 as template, 3 substitutions (G252S, Q266L and Q266P) were generated as described above, as well as 2 insertions of 12 (EU449768) and 10 residues (AY853944) respectively between aa 266 and 267. Products containing the required insertions were obtained by gene synthesis (Geneart, Life technologies, see Table 2) and sub-cloned into phrGFP-1 plasmid using *Spe*I and *BsmBI* sites. From the SK779/06 segment 6 template, 1 mutant HE harbouring an HPR2 deletion [59] was designed using a Q5 site directed mutagenesis kit (New England Biolabs) as per the manufacturer’s protocol. All constructs encoding the mutant HE and F proteins were propagated as described previously [33].

### Cell culture and transfection

Chinook salmon embryo cells (CHSE-214, ATCC 1681) were cultured, and transfected as described previously [33] using a Neon 10 μl kit (Invitrogen), and a total of 2 μg DNA per reaction (0.5 μg for HE and 1.5 μg for the F protein). Reactions were subjected to electroporation conditions of two 20 ms pulses of 1300 V and added to 3.3 ml of culture media. The same cell solution was dispensed in different culture plates including 96 and 48 well plates and 8-well chamber slides (BD Falcon). These cell monolayers were incubated for 48 h at 20°C and used in the following assays.

### Quantification of HE and F proteins surface expression by fluorescent microscopy

Monolayers cultured onto 8-well chamber glass slides were used to measure the expression of HE and F proteins at the surface of transfected cells. This was achieved using a previously described dual antibody staining method on the surface of living cells [10] with the HE labeled in green (Alexa fluor 488, Invitrogen) and the F protein in red (Alexa fluor 594, Invitrogen). Three photos of the transfected monolayers were taken for each group using an Axio Imager M2 microscope (Zeiss) at a 10x magnification and under green and red fluorescence conditions. The intensity mean values of green and red pixels were measured for each photo using ZEN 2012 image analysis software (Zeiss) and compared between different groups. A two way analysis of variance was performed on the logged fusion data, using the statistical R package (www.R-project.org, 2012).

### Content mixing assay

The content mixing assay was performed under normal culture conditions and with additional exposure to trypsin and low pH as described previously [10]. Cell monolayers expressing the HE and F-Nevis proteins were also cultured in the presence of 0, 0.1, 0.2 and 0.4 μM of calcium ionophore A23187 (Sigma) in both calcium free and calcium containing media. Final results were expressed as Firefly luciferase (FF) levels and corresponded to the average of independent triplicate experiments, each including 3 measurements. Statistical analysis was performed as described above.

### Protein fractionation

CHSE-214 cells were cultivated on 25 cm^2^ culture flasks and membrane embedded glycoproteins extracted using a Sub cellular protein fractionation kit (Thermo Scientific) as per the manufacturer’s protocol. Protein concentrations from each membrane fraction were measured using a ND 1000 nanodrop (NanoDrop Technologies, Thermo Fisher).

### Western blotting

Samples were adjusted to 10 μg of protein and mixed with 25 μl of loading buffer supplemented with beta-mercaptoethanol (1/20) (Sigma) and heated for 5 min at 95°C. Proteins were separated by SDS-polyacrylamide gel electrophoresis (SDS-PAGE) using 12% pre-cast gels (Amersham) and transferred onto a nitrocellulose membrane (VWR) by electroblotting. The membrane was treated and blocked using x1 casein solution (Vectastain) for 5 min at room temperature followed by an overnight incubation at 4°C with anti-F polyclonal antibody solution (1/500) [9] with gentle mixing. After rinsing, the membrane was incubated for 1 h at room temperature with a zebra fish anti-actin-beta monoclonal antibody solution (1/1000, AnaSpec) to act as an internal loading control. The nitrocellulose membrane was rinsed three times in blocking solution and incubated with Biotinylated anti-rabbit IgG antibody solution (1/1000) (Vectastain) followed by rinsing, and addition of a horseradish peroxidase solution (1/500) (Vector Lab) each for 1 h at room temperature. Blots were stained using ECL Western Blotting Detection Reagents (Amersham) and visualised with the C-Digit Blot Scanner (Lico Biosciences). The software Image Studio Digits 4.0 (Lico Biosciences) was used to quantify the intensity of each blot. Results were corrected against the intensity of the actin-beta controls and expressed as percentages relative to the wild type (WT) HE and F proteins. Percentages illustrate the influence of the F protein mutations or the HE HPR deletion on the proteolytic cleavage activity.

## Results

### Construction and expression of mutant ISAV glycoproteins

**Fig 1:**
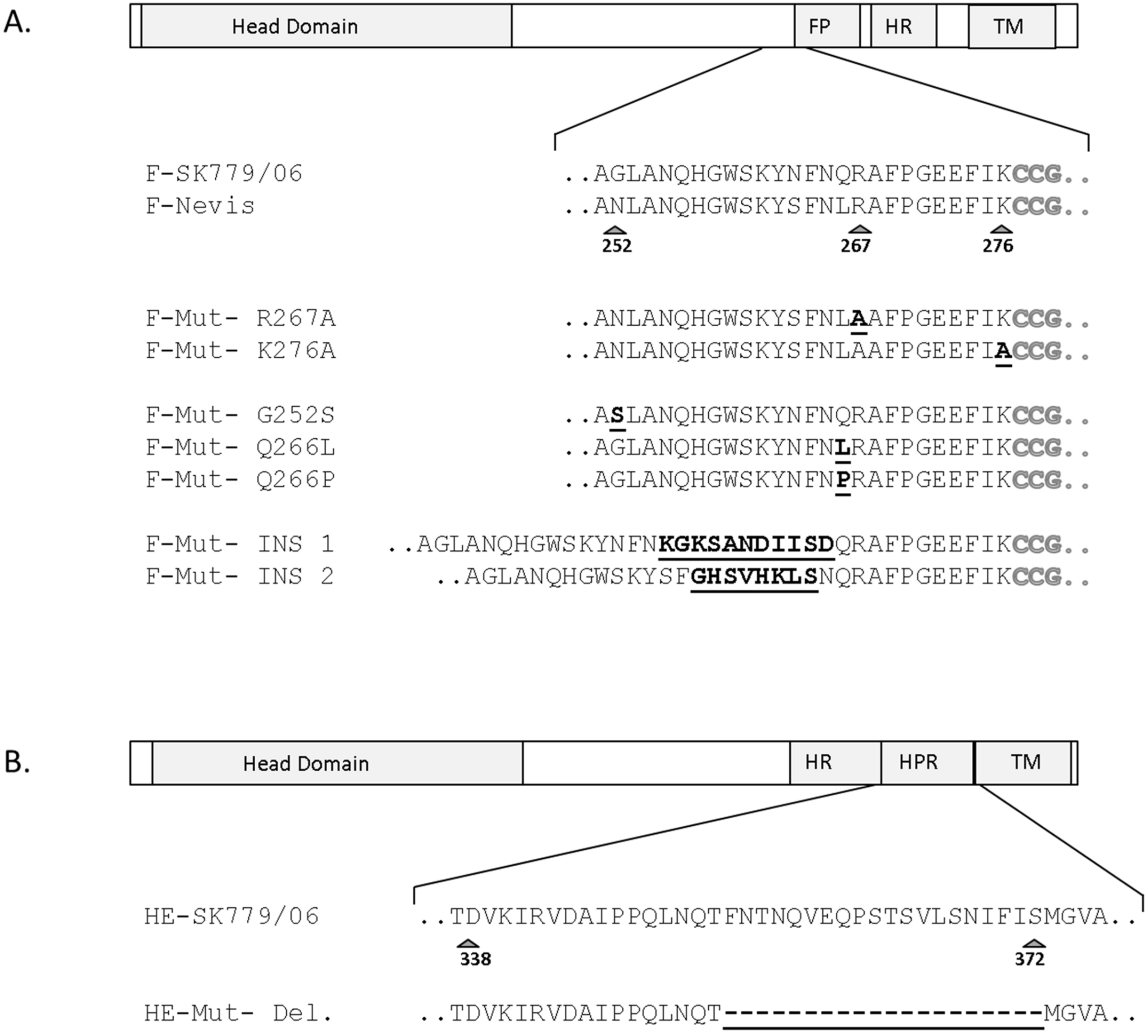
Schematic illustration of the structure of ISAV surface glycoproteins. The F protein contains the predicted transmembrane domain (TM), heptad repeat (HR) and fusion peptide (FP) which is also highlighted in grey in the sequence (A). The HE contains a Highly Polymorphic Region (HPR) (B). HRs positions were predicted using the program LearnCoil-VMF (http://cis.poly.edu/~jps/matcher.html) (data no shown). Arrows indicate important aa positions in the proteins, while substitutions and insertions introduced into the mutant F proteins (A) and the deletion in the mutant HE (B) are in bold and underlined.

Available information from previous ISAV studies [9,10] and multiple sequence alignments (data not shown) were used to generate constructs encoding several mutated F proteins and one HPR deleted HE (Fig 1). The first step involved assessing the expression level of each surface glycoprotein by immunofluorescence to verify that mutations introduced did not negatively affect glycoprotein folding and transport through the secretory pathway to the cell surface. All 7 mutant F proteins and the HPR-deleted HE mutant were expressed at levels similar to those of their respective WT homologues (Table 3). Differences in surface expression levels of each glycoprotein were not statistically significant (Table 3). Data files are provided as supplementary information (S1 File).

**Table 3:** Quantification of glycoprotein surface expression. The expression levels of each mutant glycoprotein were measured using dual antibody staining on living cells, fluorescent microscopy and image analysis. Results included measurements from cell monolayers. They were obtained from triplicate measurements, from three separate experiments and expressed as intensity mean values for green and red pixels. Expression levels of each mutated protein was compared with that of the corresponding wild type (WT) protein (in bold). Differences in surface expression levels were not statistically significant (P > 0.05).

Glycoprotein	Mutant	Intensity Mean value	Standard error (±)
Haemagglutinin-esterase	**HE-SK779/06 (WT)**	835.92	11.68
	HE-Mut- Del.	828.75	20.06
Fusion protein	**F-Nevis (WT)**	518.99	31.42
	F-Mut- R267A	450.19	28.42
	F-Mut- K276A	477.86	18.48
Fusion protein	**F-SK779/06 (WT)**	284.21	10.47
	F-Mut- G252S	327.83	37.57
	F-Mut- Q266L	290.24	15.59
	F-Mut- Q266P	290.11	17.02
	F-Mut- Ins. 1	290.31	14.12
	F-Mut- Ins. 2	286.98	14.5

### Amino acid K276 in the F protein is the proteolytic cleavage site

**Fig 2:**
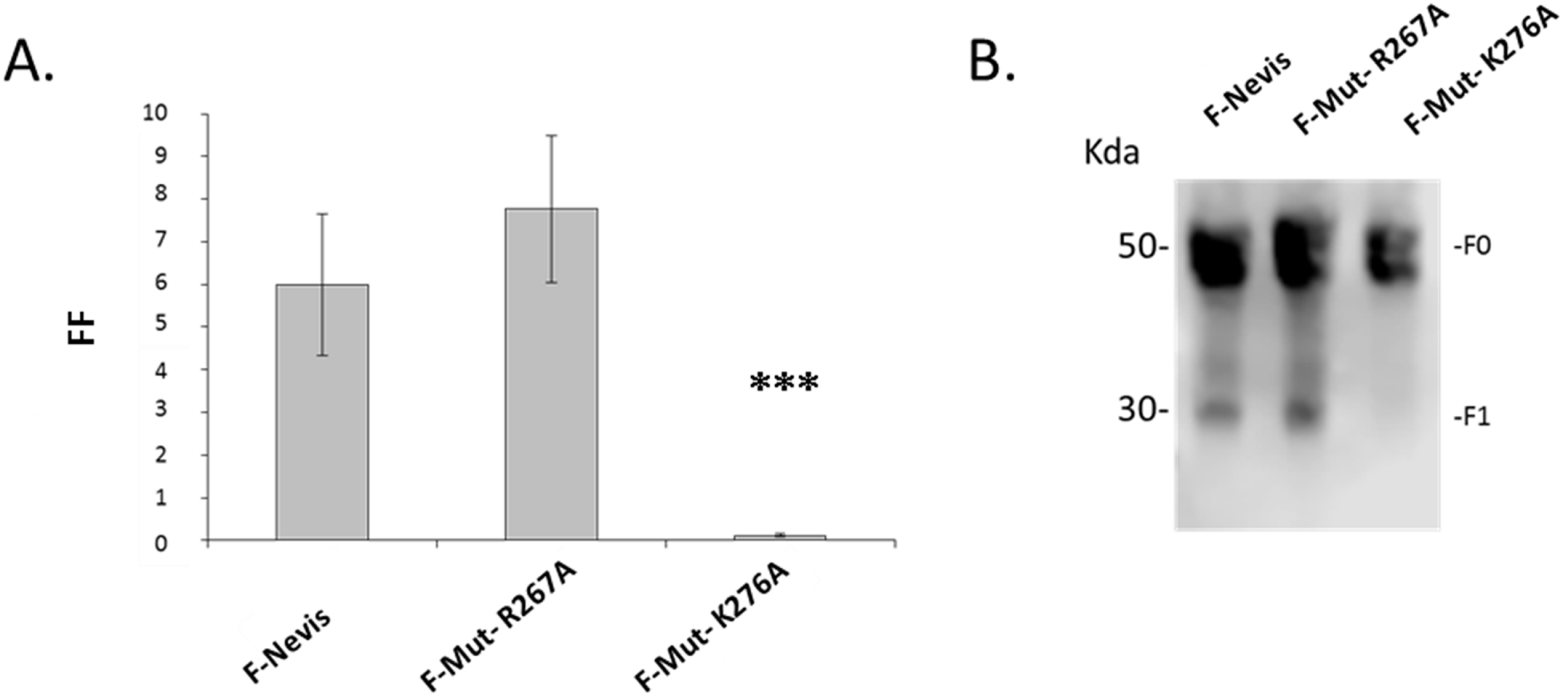
Identification of ISAV proteolytic cleavage site. Fusion activities of two mutated F proteins co-expressed with the HE Nevis were measured with a content mixing assay relying on Firefly luciferase (FF) expression (A). Proteolytic cleavage was examined by western blot using an anti F polyclonal antibody to visualise the F1 subunit band at 30 kDa (B). This blot did not include any actin-beta control. Blot intensity was assessed using the software Image Studio Digits 4.0 and results expressed as percentages relative to the WT F protein. (_***_) corresponds to P < 0.001.

Two substitutions were generated in the F Nevis protein to identify the true proteolytic cleavage site. Proteolytic cleavage still occurred in the presence of the R267A substitution as indicated by the 30 kDa F1 subunit visible in the western blot (Fig 2B, lane 2) and average fusion levels similar to those of the WT F-Nevis protein (Fig 2A). In contrast, the K276A substitution completely abrogated proteolytic cleavage as illustrated by the absence of the F1 subunit band (Fig 2B, lane 3) and the statistically significant reduction in fusion activity compare to the WT F protein (Fig 2A). Similar aa substitutions demonstrated the same findings for the F protein of the Norwegian Glesvaer isolate (data not shown). All data files for the content mixing assay are provided as supplementary information (S1 File).

### Single aa substitutions or insertions upstream of the proteolytic cleavage site enhance the fusion activity of the F protein from an HPR0 variant

**Fig 3:**
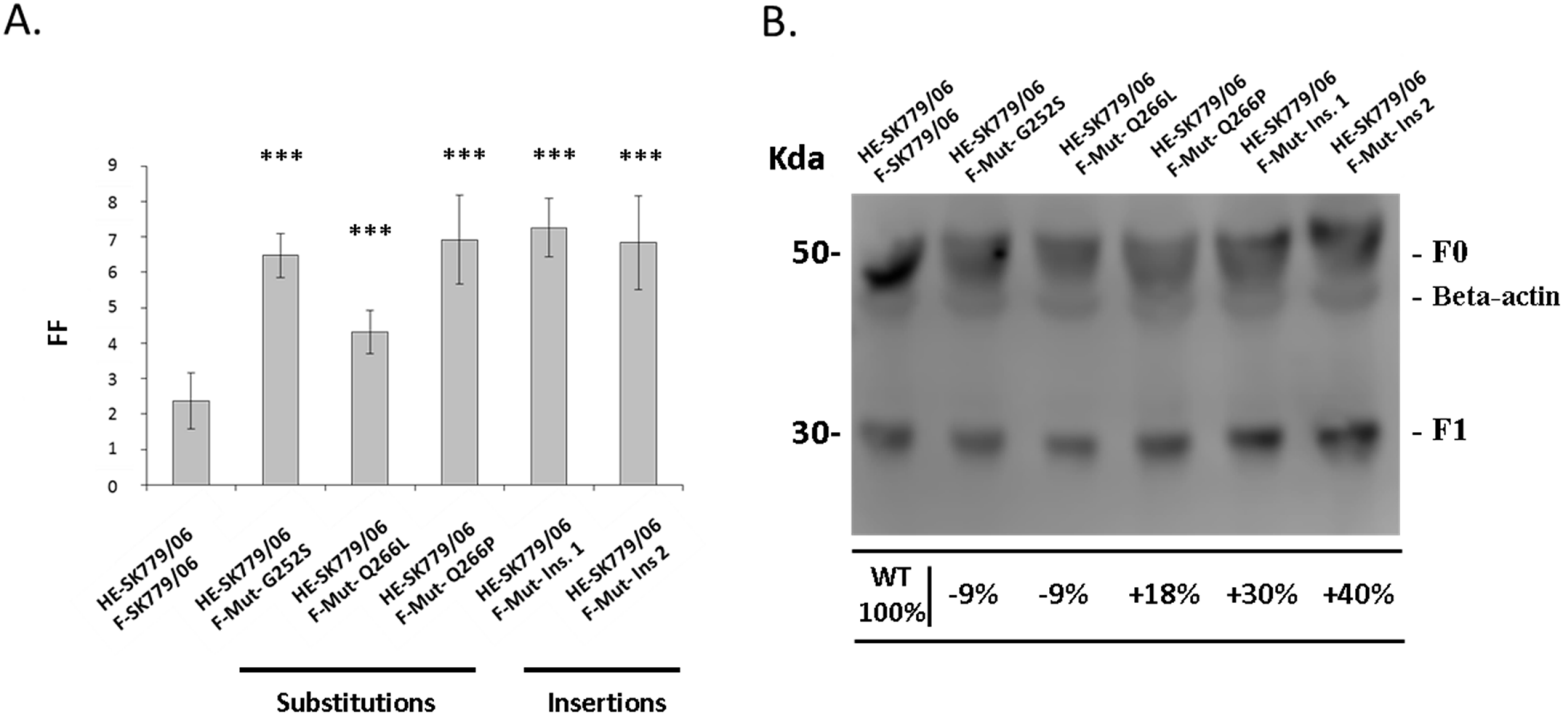
Effect of mutations upstream of the proteolytic cleavage site. Fusion activities of five mutated F proteins co-expressed with the WT HE-SK779/06 were measured with a content mixing assay (A). Proteolytic cleavage was examined by western blot (B). Blot intensity was assessed using the software Image Studio Digits 4.0 and results expressed as percentages relative to the WT F protein. Percentages illustrate the effect of F protein mutations on the proteolytic cleavage. (_***_) corresponds to P < 0.001.

Having identified K276 as the true proteolytic cleavage site, this study then set out to investigate whether single F protein mutations, in the region immediately upstream of the cleavage site, would influence fusion. For this purpose, constructs encoding 5 mutant F proteins were created from the WT F-SK779/06 protein sequence and co-expressed with the homotypic HE. All 3 substitutions (F-Mut- G252S, F-Mut- Q266L and F-Mut- Q266P) and 2 insertions (F-Mut- Ins. 1 and F-Mut- Ins. 2) enhanced significantly the fusion activity of the mutant F proteins (Fig 3A). An increase in proteolytic cleavage activity was also observed for F-Mut- Q266P, F-Mut- Ins. 1 and F-Mut– Ins. 2 compared to the WT F-SK779/06 (Fig 3B, lanes 4, 5 and 6), but not for F-Mut- G252S and F-Mut- Q266L (Fig 3B, lanes 2 and 3). Variations between experiments were observed with the content assay, but did not have any statistically significant effect on the overall fusion differences between each glycoprotein combination.

### Fusion was activated without the addition of exogenous trypsin when the F proteins were combined with an HPR deleted HE

**Fig 4:**
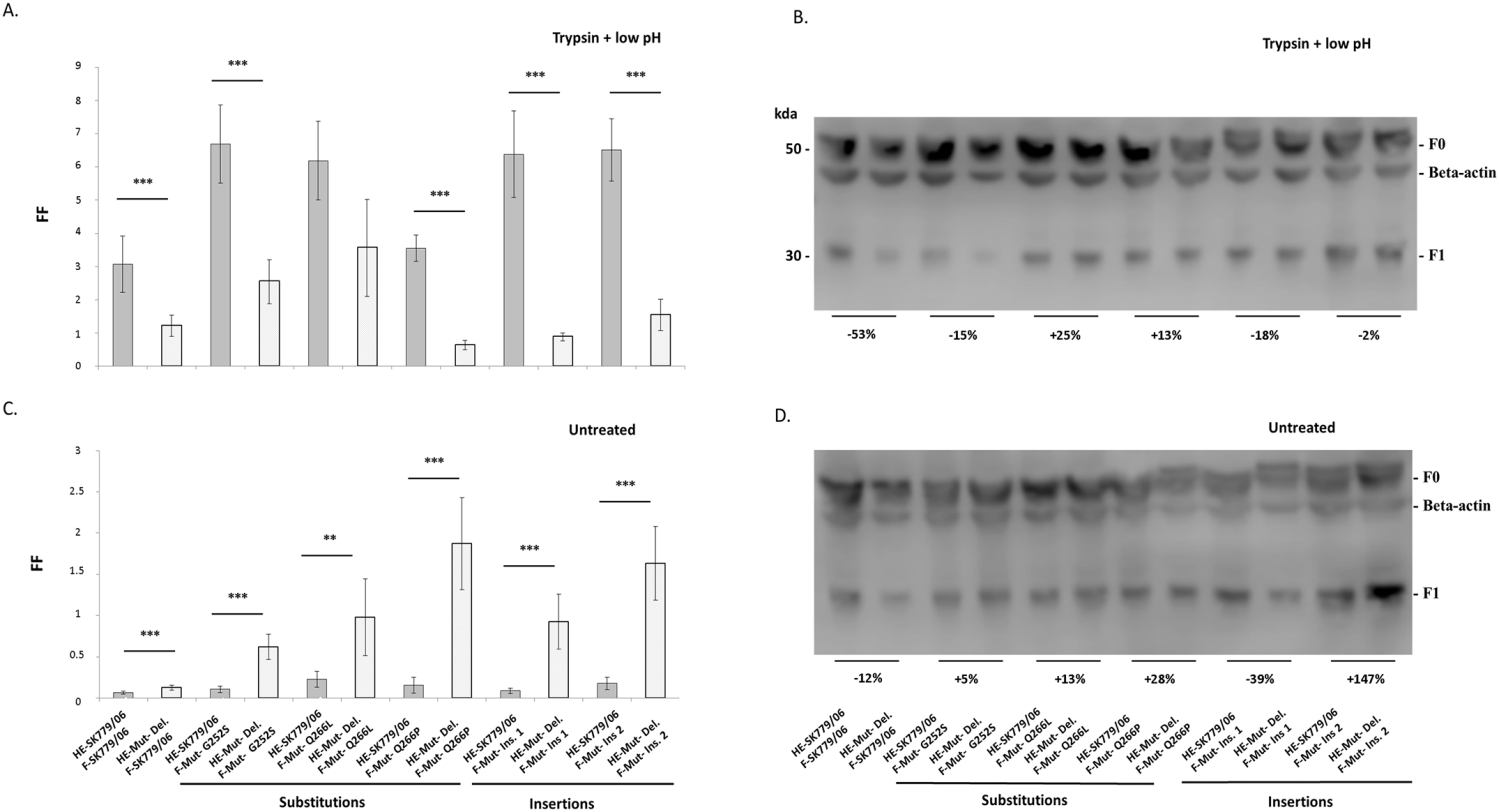
Fusion activity and proteolytic cleavage of mutant HE and F protein combinations under different culture conditions. Fusion activities of combinations of glycoproteins containing either a full length HPR HE (A and C, grey bars) or a deleted HPR HE (A and C, white bars) co-expressed with mutated F proteins were measured with a content mixing assay under two culture conditions: with trypsin and low pH treatments applied to the cell monolayer (A) and under normal culture conditions (C). Proteolytic cleavage of mutant HE and F proteins under the two same culture conditions was examined by western blot (B and D). Percentages under each pair of blots were obtained by comparing the blot intensity of the group containing a deleted HPR HE and the one from the WT HE SK779/06 with a full length HPR. Therefore, percentages illustrate the effect of the HPR deletion on the proteolytic cleavage. (_***_) corresponds to P < 0.001 and (_**_) to P < 0.01.

In the knowledge that F protein mutations and HE HPR deletions [10] independently influenced viral fusion, the next step was to investigate the combined effect of these different mutations. For this purpose, an additional HE mutant with an HPR deletion was generated from the WT full length HE-SK779/06 and co-expressed with the different mutated F proteins based on the F-SK779/06 protein. Fusion activity was assessed following addition of exogenous trypsin and low pH (Fig 4A) and under normal culture conditions (Fig 4C).

All the glycoprotein groups containing a full length HPR HE had a significantly higher fusion activity when exogenous trypsin and low pH were applied (Fig 4A, in grey), compared to the corresponding groups with a deleted HPR HE (Fig 4A, in white). Under these culture conditions 4 out of the 6 groups of glycoproteins with a full length HPR HE also demonstrated a higher proteolytic cleavage activity than those of the corresponding groups with an HPR deleted HE, as illustrated by the decreases in percentages (Fig 4B). In complete contrast to the above results, the same groups containing a full length HPR HE had a poor ability to trigger fusion when the cell monolayers were left untreated (Fig 4C, in grey), while the groups consisting of an HPR deleted HE were capable of moderate but significantly higher levels of fusion, despite the lack of exogenous trypsin and low pH in the culture medium (Fig 4C, in white). In the absence of exogenous culture treatments, 4 out of the 6 groups of glycoproteins with a deleted HPR HE also displayed a higher proteolytic cleavage activity than those of the corresponding groups with a full length HPR HE, as illustrated by the increases in percentages (Fig 4D). As in previous experiments (Fig 3), combined F protein mutations resulted in higher fusion levels than with the initial WT F-SK779/06 protein (Fig 4A and 4C).

Additional observations were also made with the untreated cell monolayers. Two days post-haemadsorption, unaltered Atlantic salmon RBCs tended to detach easily from the cell monolayers transfected with a combination of glycoproteins containing a full length HPR HE. In contrast, RBCs remained firmly attached to the cells expressing F proteins co-expressed with a HPR deleted HE, confirming that the erythrocytes membranes were fused with those of the transfected cells. Measurements of haemoglobin content [60] confirmed these observations (data no shown).

### Calcium ionophore A23187 inhibits ISAV fusion in a dose dependent manner

**Fig 5:**
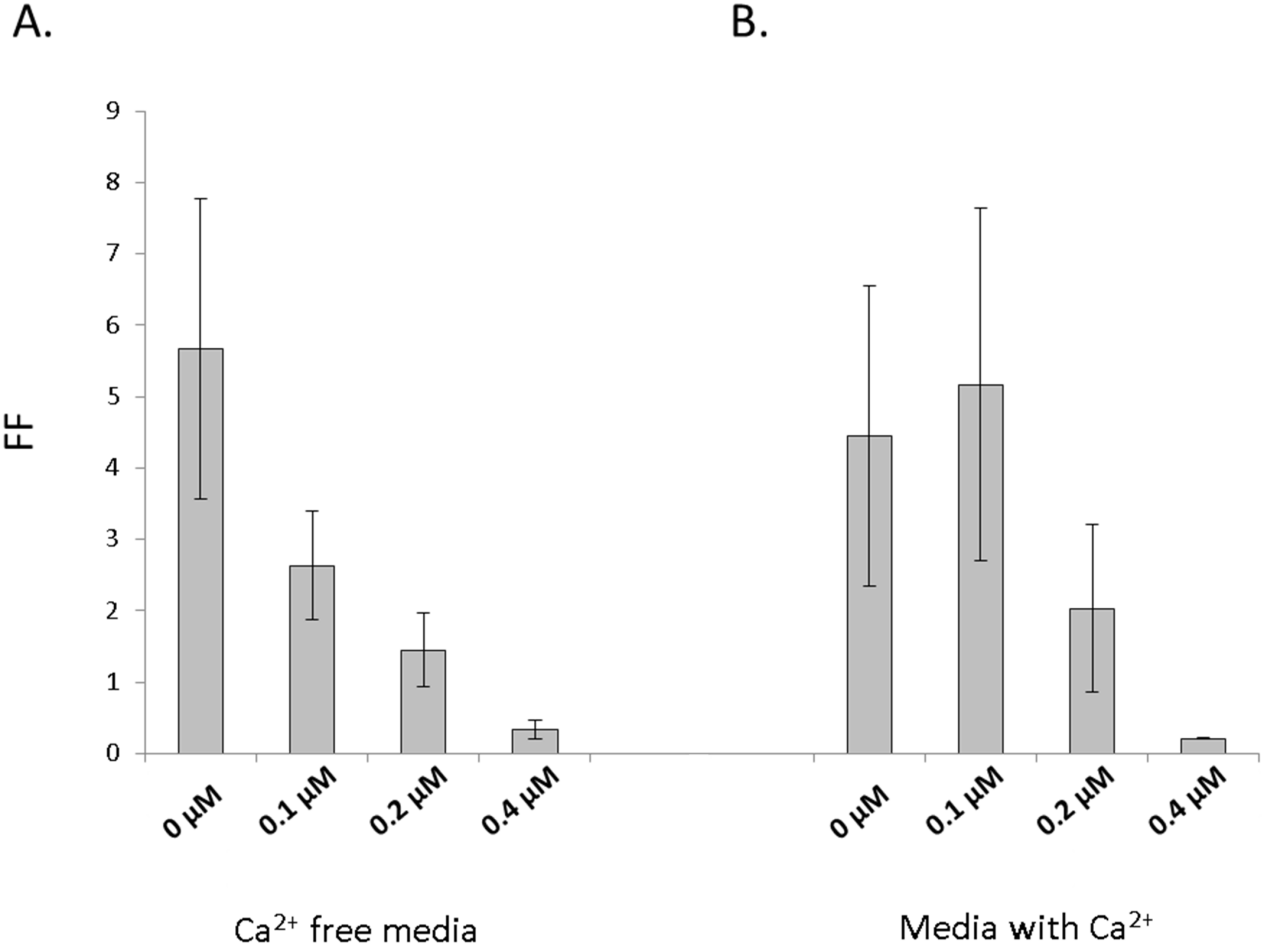
Fusion activity of HE and F-Nevis protein with addition of calcium ionophore A23187 to the culture media. Fusion activity of the WT HE and F-Nevis protein was measured as described before in the presence of various concentrations of calcium ionophore A23187 in calcium free (A) and calcium containing media (B) to examine if the activating protease was calcium dependent.

Finally, in an effort to narrow down the type of host cell proteases potentially involved in ISAV fusion, untreated cell monolayers expressing the HE and F-Nevis protein were cultured with various doses of calcium ionophore A23187 and fusion measured. Fusion levels detected in the absence of A23187 confirm previous findings that HPR deleted HE from a WT pathogenic strain co-expressed with its homotypic F protein can trigger fusion without the addition of exogenous trypsin (Fig 5). Reduction in fusion activity was dose dependent and was particularly noticeable and gradual in the calcium free media (Fig 5A). A decrease in fusion activity was also recorded in media containing calcium, but only for the two highest calcium ionophore A23187 concentrations (Fig 5B). These results suggested that the activating protease for ISAV fusion was calcium dependent.

## Discussion

This functional analysis demonstrated how specific mutations in both the HE and F proteins from an ISAV HPR0 genotype enhance viral fusion. These mutations appeared to influence different steps of the fusion process and regulate fusion through various mechanisms. Certain substitutions and insertions upstream of the proteolytic cleavage site promoted cleavage activity, while others appear to influence post-cleavage steps. In comparison, HPR deletions influence the way the HE primes the F protein and facilitates either cleavage by an ubiquitous host cell protease and/ or post-cleavage refolding of the F protein. As in other orthomyxoviruses and paramyxoviruses, ISAV surface glycoprotein mutations shape the interaction between the attachment and F protein which ultimately influences viral fusion. This may in part contribute to the different degree of virulence and tissue/cell tropism reported for HPR0 and HPR deleted ISAV genotypes.

After having confirmed that the mutations did not influence the surface expression of the mutant glycoproteins, the first objective was to identify the proteolytic cleavage site as R267 and K276 have both been proposed previously to carry this feature [9]. As well as clarifying a basic function of the ISAV F protein, this identification of the cleavage site helped to establish a reference point from which all subsequent F protein mutations were generated. These were all located upstream and in close proximity to the cleavage site. Similarly positioned mutations influenced the proteolytic cleavage and fusion function in influenza A [19,61] and Hendra virus [62].

In this first set of experiments (Fig 2), fusion was triggered with exogenous trypsin. This pancreatic serine protease is highly specific and reactive to the positively charged side chains of Lysine (K) and Arginine (R) residues [63]. The active site of trypsin consists of a catalytic triad: H57, D102 and S195 which cleaves the peptide at the carboxyl side of the aa side chain using a hydrolytic reaction [64–66]. The R267A substitution in the F protein did not prevent proteolytic cleavage and fusion to occur therefore implying that the hydrolytic reaction carried out by the trypsin active site was still occurring elsewhere on the protein. In contrast, the K276A substitution completely prevented both the cleavage and fusion. An alanine is a neutral and particularly innocuous residue as it carries a non-polar side chain [67]. This non-reactive side chain prevented key steps of the hydrolytic reaction to occur and therefore stop proteolytic cleavage and subsequent fusion. This provided strong evidence that K276 is the true proteolytic cleavage site in the ISAV F protein. This is further supported by the fact that K276 is located immediately upstream of the N-terminus of the proposed fusion peptide region delimited by C277 and Y292, [9], a position similar to the proteolytic site of other orthomyxoviruses [68,69], coronaviruses [70] and paramyxoviruses [71,72].

In this study, each mutation type was designed to influence a specific stage of the fusion process. The F protein mutations located in close vicinity to the cleavage site were expected to impact the proteolytic cleavage and the conversion of the F protein into its F1-F2 biologically active conformation [73]. The G252S substitution was selected due to the high degree of variability at this particular position [58]. A serine was present at position 252 only in a few ISAV isolates [58]. The Q266L has been the subject of a more thorough study and has been reported as a potentially important virulence marker since the majority of HPR0 variants carry a Q at this position as opposed to a L for most virulent strains [56]. Only few strains of ISAV were reported with a P at position 266 [74]. All these 3 substitutions significantly increased viral fusion, but only Q266P enhanced the proteolytic cleavage. Although this study did not directly investigate the structural effect of these substitutions, it is possible that they may alter the shape and refolding of the F protein in such a way that the exogenous trypsin could more readily access the cleavage site. In avian influenza viruses, variations in the 3 aa immediately upstream of the cleavage site greatly influence the cleavage by trypsin in different HA subtypes [69], with certain substitutions making the cleavage loop more exposed and readily accessible to host proteases [30,69,75].

Acquisition of extra sequences through recombination of the HA gene is well documented in influenza viruses [76–78]. In ISAV, insertion 2 (AY853944) occurred through recombination of the F gene with a partial sequence of segment 3 [58], and insertion 1 (EU449768) with a part of segment 2 [36]. These two insertions resulted in the addition of 1 and 2 extra K residues which could act as potential new productive cleavage sites. Addition of extra active trypsin cleavage sites has been reported for the highly pathogenic H7N3 avian influenza strain [79] and similar observations were made with the highly virulent Chilean isolate ISAV3-89 [80]. In the same study, modelling of the F protein from other ISAV isolates predicted that F protein insertions can also increase the number of recognition sites for other endopeptidases [80]. Another possibility is that like in human [81] and avian influenza [79], insertions in this region of the F protein may structurally extend the cleavage loop, making it more prone to enzymatic processing. Protein modelling of the ISAV F protein demonstrated that insertions, similar to those used in this study, are flexible regions which could promote access to the proteolytic processing site [80].

In paramyxoviruses, the stalk of the attachment protein is involved with the activation of the F protein [31,47,82]. Upon receptor binding, structural changes are propagated from the attachment protein globular head down to the stalk [83]. This reconfigures the stalk and transfers energy which alters the stability of the F protein thereby triggering structural rearrangements that expose the cleavage site [84–86]. Therefore, the mutant HPR0 HE with a deleted HPR provided information specifically on this early fusion triggering phase where the HE primes the F protein prior to cleavage. Fusion results under different culture conditions (Fig 4) suggest that the HPR length influences how the HE interacts and activates the F protein, consequently altering subsequent fusion. A possibility is that the HPR length may influence the structural rearrangement of the HE stalk following receptor binding. In paramyxoviruses, engineered disulphide bonds and mutations can disrupt the haemagglutinin stalk flexibility, which impede tetramer dissociation and inhibit the triggering of fusion [82,87].

Parallels drawn with low and high virulence influenza [48–51,88] and NDV strains [52,53] may help understanding of the reasons behind the differences in fusion activity between the HPR0 and deleted HPR HE. The length of the HE HPR may indirectly influence how the F protein is cleaved. A full length HPR0 HE could potentially have difficulties in priming and activating the F protein. Previously, full length HPR HEs were found to induce lower levels of HE and F protein separation following receptor binding and had a reduced fusion activity [10]. This could potentially prevent intracellular refolding of the F protein, making the cleavage site inaccessible to ubiquitous cellular endoproteases while in transit through the host Golgi apparatus and/or trans-Golgi network [89]. Results in Fig 4D confirmed that most of the glycoprotein groups with a full length HPR HE induced lower level of proteolytic cleavage than their deleted counterparts without any exogenous culture treatments. This suggested that ISAV F proteins may have reached the surface in an uncleaved, inactive form. A similar situation has been reported for NDV, although intracellular cleavage is limited by the presence of a single basic residue at the cleavage site [46,53]. This leads to the F protein being cleaved and fusion triggered only when trypsin and low pH are applied at the cell surface [53,90], as observed in this study (Fig 4A). This would explain why the same ISAV F proteins coupled with a full length HPR HE did not show any fusion activity when cell monolayers were left untreated (Fig 4C).

In contrast, when associated with a deleted HPR HE, a F protein may be more easily activated and prone to proteolytic cleavage. Previous work supports this as HPR deleted HEs were shown to induce higher levels of HE and F protein separation post-receptor binding and were associated with a greater fusion activity [10]. Results in Fig 4D indicated that most of the glycoprotein groups with a deleted HPR HE led to higher level of proteolytic cleavage than their full length counterparts in the absence of exogenous culture treatments. This suggested that the majority of F proteins may have been cleaved intracellularly and reached the cell surface in a pre-activated state and already capable of moderate levels of fusion, even at neutral pH. Similarities can be drawn with NDVs possessing polybasic recognition sites which enable their F proteins to be cleaved by furin in the trans-Golgi membrane, that then reach the cell surface cleaved and pre-activated [53,91,92]. However, furin is unlikely to be the host enzyme responsible for ISAV proteolytic cleavage, since the F protein does not harbour any typical furin recognition sites (data not shown). When administered to the calcium-free medium, calcium ionophore A23187 reduced fusion activity by inactivating the cleavage enzyme through depletion of intracellular calcium [89,93]. This indicated that the Atlantic salmon protease involved in the processing of ISAV F protein was calcium dependent and that it may belong to the proprotein convertase family [94–96] or equivalent in teleost fish.

However, the present results also suggested that the variations in fusion activities (Fig 4A and 4C) could not all be directly attributed to differences in proteolytic cleavage abilities (Fig 4B and 4D), indicating that HE mutations may alter other underlying mechanisms of the fusion process. An alternative explanation could be that instead of influencing cleavage, HPR length affects subsequent refolding steps which lead to the F protein adopting its post-fusion conformation. Previous work has demonstrated that in certain SV5 F proteins, cleavage did not necessarily result in fusion activity and syncytia formation [97]. ISAV F protein refolding is triggered by low pH as demonstrated by fusion assays [9,10]. In other viruses, this stimulus induces major F protein structural rearrangements and the assembly of the head-domain heptad repeats into a central, trimeric α-helical coiled coil structure that propel the fusion peptide towards the target membrane [84,98]. Recent structural analysis confirmed that in ISAV F protein, residues E327 and E329 carboxyl-carbonate acts as a pH sensor that once activated at low pH binds and stabilises the extended central coiled-coil region via strong short hydrogen bonds [45]. In the present study, both the full length and deleted HPR HE could potentially allow the proteolytic cleavage to take place. However, when associated with a full length HPR0 HE, the poor activation of the F protein may imply that post-cleavage refolding could only be achieved with additional stimulus in the form of low pH treatment applied at the cell surface. In comparison, the high level of F protein activation induced by an HPR deleted HE may facilitate post-cleavage structural rearrangement that could be more easily achieved at neutral pH in the untreated cell monolayers.

Both the proteolytic activation of the F protein and the type of host protease involved, play a significant role in relation to the functional differences between HPR deleted and HPR0 ISAV genotypes. Therefore, like in influenza viruses and NDV, ISAV fusion is an important determinant of virulence and together with other factors, such as receptor availability on the cell surface [99], interferon antagonistic properties [100–102] and certain polymerase features [103], partly contributes to the varying degree of virulence reported for different HPR genotypes. Variation in fusion ability linked to HE HPR deletions and F protein mutations may also explain the different tissue [4] and cell tropism reported in ISAV [15,99,104]. We propose that the fusion protein of viruses carrying full length HPR HE may be mainly activated by trypsin-like proteases, most likely at the cell surface. Some of these enzymes are principally found in the mucosal surfaces [105,106] and therefore would limit the replication of the HPR0 variant to these tissues, causing only mild and sub-clinical infection in gill as reported by field data [4]. In contrast, viruses carrying an HPR deleted HE may have their F protein activated by ubiquitous host cell proteases present in a wider array of tissues. This would allow viruses to fuse and replicate in a wider range of cells and tissues and cause a systemic infection that may lead to mortality, as corroborated by field data [107].

The present results demonstrate how the combined effect of an HPR deletion in the HE and specific mutations in the F proteins can enhance viral fusion. Both substitutions and insertions close to and upstream of the cleavage site increased viral fusion. An HPR deletion influenced the ability of HE to prime the F protein and promote fusion activation by an ubiquitous host protease and/or subsequent post-cleavage refolding steps. Future work is still needed to fully characterise the fusion mechanisms affected by these mutations and to specifically identify the host protease involved in ISAV fusion. These findings emphasise that the variation in fusion ability between HPR0 and HPR deleted ISAV genotypes may be one of the factors contributing toward the difference in virulence and tissue/cell tropism.

## Supporting Information

S1 File Excel spread sheet providing the raw data for the surface expression of the HE and F protein and for the content mixing assay. (XLSX)
